# Supplementary figures and images for: Cross-species insemination reveals mouse sperm ability to enter and cross the fish micropyle
Source: eLife. 2025 Oct 16;14:RP106303. doi: 10.7554/eLife.106303 (PMC12530800; doi:10.7554/eLife.106303)

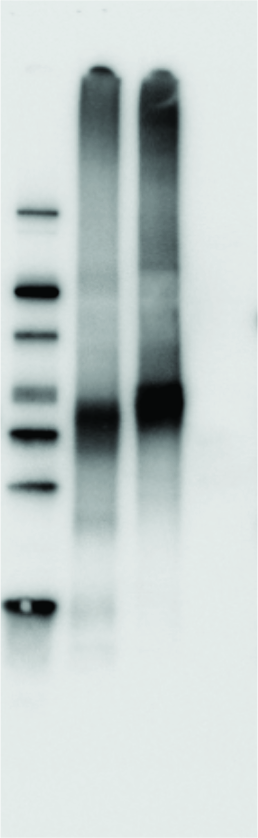

Supplement: Figure 1—source data 1. [file elife-106303-fig1-data1.zip › SourceData_Fig1B_UNlabeled.tif]

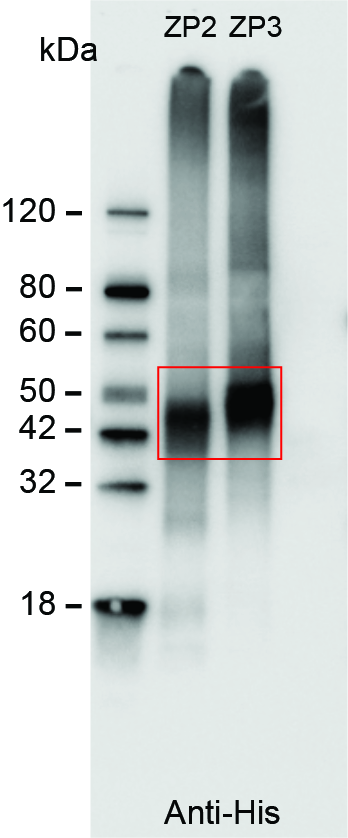

Supplement: Figure 1—source data 2. [file elife-106303-fig1-data2.zip › SourceData_Fig1B_labeled.tif]
